# Plasma Cholinesterase Activity in Patients With Rheumatoid Arthritis and Toxoplasmosis

**DOI:** 10.7759/cureus.50979

**Published:** 2023-12-23

**Authors:** Rabie G Abdullah, Souzan H Eassa, Fouad K Mohammad

**Affiliations:** 1 Department of Pharmacology, College of Pharmacy, University of Duhok, Duhok, IRQ; 2 Molecular and Microbiology Division, School of Medicine, University of Kurdistan Hewlêr, Erbil, IRQ; 3 Department of Physiology, Biochemistry and Pharmacology, College of Veterinary Medicine, University of Mosul, Mosul, IRQ; 4 Pharmacology and Toxicology, College of Nursing, the American University of Kurdistan, Duhok, IRQ

**Keywords:** anti-rheumatoid drugs, biologic therapy, dichlorvos, cholinesterase, toxoplasma gondii, arthritis

## Abstract

Background and objective

Rheumatoid arthritis (RA) is a chronic autoimmune disease causing synovium inflammation and functional impairment. Toxoplasmosis is an intracellular zoonotic parasitic infection and a risk factor in immunosuppressed diseases including RA. The involvement of the cholinergic mechanism is not clear when both diseases exist in combination. This study aimed to examine plasma cholinesterase (ChE) activity in patients suffering from RA with concomitant toxoplasmosis, taking into account the enzyme susceptibility to in vitro inhibitory challenge with the organophosphate dichlorvos in RA patients.

Methods

This was a case-control study involving 88 RA patients and 61 healthy controls of both genders. The RA patients were allocated into three groups. The first group received no therapy (n=14), the second group received conventional anti-arthritis therapy (n=49), and the third group received conventional + biologic therapy (n=25). Plasma ChE activity was determined by an electrometric method. Plasma samples were screened for *Toxoplasma gondii (T. gondii)* infection, using ELISA *T. gondii* antibodies IgG and IgM. In vitro inhibition of plasma ChE activity was assessed by incubating the samples with dichlorvos at 0.25 and 0.5 μM. The time-dependent dichlorvos (0.25 μM)-induced plasma ChE inhibition and its kinetics were determined.

Results

The RA patients comprised 76 (86.4%) females and 12 males (13.6%), whereas healthy controls included 22 (36.1%) females and 39 (63.9%) males. The rates of toxoplasmosis IgG positivity in controls and RA patients were 26.2% and 39.8%, respectively. Plasma ChE activity in patients with RA was significantly higher than that in the control group, by 16%. Plasma ChE values of RA patients with conventional therapy and conventional + biologic therapy were higher than that of the control group, by 18% and 27%, respectively. Odds and risk ratios of elevated plasma ChE activity (20%) in RA patients with therapy indicated that high plasma ChE activity among RA patients with therapy is a risk factor. The plasma ChE activity of *T. gondii* IgG-positive RA patients was not significantly different from that of the IgG-negative ones. Dichlorvos at 0.25 and 0.5 μM significantly inhibited in vitro plasma ChE activity in controls and RA patients. The rates of plasma ChE inhibition by dichlorvos were lower in the RA groups with conventional therapy in comparison with those in the control group (77% vs. 91%). Examining the dichlorvos time-dependent ChE inhibition kinetics, RA groups showed increases in the half-life of inhibition by 23.6% to 32.7% and the total inhibition time by 23.5% to 32.5%, together with decreases in the inhibition rate constant by 19% to 24.5%, an indication of reduced inhibition rate of plasma ChE activity compared to that of the control group.

Conclusions

The autoimmune nature of RA and its chronicity might have contributed to the increase in plasma ChE activity among the patients. This increase in enzyme activity could be a risk factor in RA patients undergoing conventional therapy alone or in combination with biologic therapy; however, the clinical significance of such a condition remains obscure at present. The in vitro inhibition of plasma ChE activity in RA patients suggests reduced susceptibility of the enzyme to ChE inhibition by dichlorvos. Toxoplasmosis was not a risk factor when plasma ChE activity was taken into account among RA patients.

## Introduction

Rheumatoid arthritis (RA) is an autoimmune disease characterized by chronic synovium inflammation and functional impairment due to cartilage and bone destruction of the joints [1,2]. The disease affects about 1% of the population globally, predominantly females [3]. Treatment protocols for RA vary considerably according to the signs and symptoms of the disease, the nature of the lesions on the joints, and the chronicity of the condition [4]. The usual therapeutic agents used in RA patients include but are not limited to non-steroidal medications, conventional (disease-modifying) anti-rheumatoid drugs as well as biologic agents due to the autoimmune response nature of the disease resulting in the release of pro-inflammatory cytokines [5-7].

*Toxoplasma gondii (T. gondii) *is a zoonotic protozoan parasite that causes toxoplasmosis, which is an intracellular infection prevalent in about one-third of the human population [8]. The susceptibility to toxoplasmosis is especially high in immunocompromised patients [8-10]. Being a risk factor, toxoplasmosis is identified as an increased positivity rate of anti-*T. gondii *IgG, which is found to be highly associated with RA in patients, with the possibility of exacerbating the latter condition [11-13]. Due to the immune-pathogenic nature of RA and the possible complications in terms of its association with toxoplasmosis, activation of inflammatory/anti-inflammatory responses might occur, such as the production of cytokines (interleukin-12 and interferon-gamma) as well as reactive nitrogen and oxygen species [12,14].

Cholinergic mechanisms involving blood and tissue cholinesterase (ChE) activities have been identified in inflammatory conditions [15,16]. High ChE activity was found in low-grade, slowly developing inflammatory diseases, and such conditions might modulate cholinergic neurotransmission and immune response [15]. Modulation of blood ChE has been observed in patients with RA with no clear-cut increase or decrease in activity [16,17]. Furthermore, as the cholinergic anti-inflammatory pathway was implicated in cognitive functions in toxoplasmosis [18], potential changes in ChE activity among RA patients with toxoplasmosis have not been explored clinically. Studies on ChE activity have suggested increased plasma and lymphocyte ChE activities in rats [19] and neuronal ChE activity in mice [20,21] experimentally infected with *T. gondii*. However, another study has reported decreased ChE activity in mice acutely infected with *T. gondii,* probably due to acute liver injury [22]. Within this context and in light of scarce information on ChE activity in patients with RA and toxoplasmosis, especially when they exist in combination, the present study sought to examine plasma ChE activity in patients suffering from RA with or without concomitant toxoplasmosis in the city of Duhok, Kurdistan Region, Iraq. Another aspect of the present study was the attempt to challenge the plasma ChE of selected RA patients by the in vitro inhibition technique, using the organophosphate dichlorvos, in order to examine the enzyme susceptibility to the inhibition, and whether the observed changes in plasma ChE in RA patients can modify the response to such an inhibition.

## Materials and methods

Patient selection and inclusion criteria

This was a case-control study conducted to determine plasma ChE activity and screen the plasma for toxoplasma infection in patients with RA in Duhok, Kurdistan Region, Iraq, from February 2022 to June 2023. A total of 88 patients of both genders with RA were recruited, regardless of concurrent therapy with anti-arthritic medications, from the Duhok Center for Rheumatic Diseases and Medical Rehabilitation, Duhok, Iraq. RA was diagnosed and classified by specialists in rheumatology at the center based on the fulfillment of the 2010 criteria of the American College of Rheumatology/European League Against Rheumatism (https://rheumatology.org/criteria) [23].

Accordingly, patients were considered for the RA test if they had at least one joint with clinical synovitis that cannot be attributed to another disease. Specifically, patients had to have a score of ≥6/10 (A to D) to be classified as having RA. Briefly, the scoring system was as follows: A: Joint involvement, 2-10 large joints (1), 1-3 small joints (with or without involvement of large joints) (2), 4-10 small joints (with or without involvement of large joints) (3), >10 joints (at least one small joint) (5); B: Serology results (at least one is required), low-positive rheumatoid factor (RF) or low-positive anti-citrullinated protein antibody (ACPA) (2), high-positive RF or high-positive ACPA (3); C: Acute-phase reactants (at least one test result), abnormal C-reactive protein or abnormal erythrocyte sedimentation rate (1); D: Duration of symptoms, ≥6 weeks (1). Patients suffering from RA, but with moderate comorbidities (such as uncontrolled diabetes, heart diseases, respiratory diseases, and patients on depressants and dementia therapy) to severe ones (such as cancer, COVID-19, severe pneumonia, kidney failure, and heart failure) were excluded from the study. Pregnant women were also excluded from the study.

The selected patients were allocated into three groups at the time of the interview according to the therapy they received. The first group received no therapy (n=14), the second group received conventional anti-arthritis therapy that included methotrexate, corticosteroids, and other disease-modifying anti-rheumatic medications (n=49), and the third group received conventional anti-arthritis therapy concurrently with biologic medications such as infliximab, adalimumab and etanercept (n=25). A control group consisting of voluntary healthy participants (n=61) was also included. The demographic characteristics of all participants in the study were recorded after interviewing them.

Ethical approval

The Committee of Post Graduate Studies, College of Pharmacy, University of Duhok, Kurdistan Region, Iraq, and the Research Ethics Committee, Duhok Directorate General of Health, Duhok, Kurdistan Region, Iraq provided the approval to conduct the present study (reference no. 15092021-9-14 R1) according to the guidelines of Helsinki declaration. Written consents were obtained from patients and healthy controls recruited for the study. They were briefed about the purpose of the study, the nature of data collection, the blood sampling method, and the expected study outcomes. Participants’ information was kept confidential.

Blood sampling

A certified nurse drew about 5 ml of venous blood samples from patients and controls into heparinized vacutainer tubes. Plasma was separated from the blood by centrifugation at 3000 rpm for 15 minutes and aliquots were kept at -20°C pending analysis within one month.

Determination of plasma ChE activity

We used an electrometric method to determine plasma ChE by incubating 0.2 ml of plasma aliquot with a reaction mixture containing 3 ml of distilled water, 3 ml of barbital-phosphate buffer (1.237 g sodium barbital, 0.163 g potassium dihydrogen phosphate, and 35.07 g sodium chloride/L of distilled water, pH 8.1) [24,25]. After measuring the pH1 of the mixture with the glass electrode of a pH meter (pH700, Eutech Instruments, Singapore), 0.1 ml of 7.1% aqueous solution of acetylcholine iodide was added, and the mixture was incubated in a water bath at 37 ºC for 20 minutes. Thereafter, the pH2 of the mixture was measured. Plasma ChE activity was estimated as follows:

Plasma ChE activity (Δ pH/20 min) = (pH1 - pH2) - Δ pH of blank (no plasma sample).

Screening for toxoplasmosis

Plasma samples obtained from all the participants were screened for *T. gondii* infection by using human anti-*T. gondii* antibody IgG ELISA kit (Cat. No ED0537Hu, BT LAB Co., Shanghai, China). The IgG-positive cases were also tested by the human anti-*T. gondii* antibody IgM ELISA kit (Cat. No ED0538Hu, BT LAB Co.). The Avidity *T. gondii* IgG ELISA kit (NovaLisa®, Dietzenbach, Germany) was also used to determine the contemporary status of the infection.

In vitro inhibition of plasma ChE activity by dichlorvos

Aliquots of plasma samples obtained from the controls and the three RA groups (10/group) were pooled separately. We used the technique of 10-min in vitro incubation of a ChE inhibitor (in this case the organophosphate dichlorvos) with 0.2 ml of pooled plasma aliquot to induce enzyme activity inhibition as reported before [24,25]. An aqueous solution of dichlorvos (Dichlorvos 50% EC, Nicoz, China) was freshly prepared for in vitro inhibition usage at final concentrations of 0.25 or 0.5 μM in the plasma-ChE reaction mixture (6.3 mL). These concentrations of dichlorvos have been previously used for in vitro blood ChE inhibition [25,26]. The plasma aliquot-dichlorvos reaction mixtures were incubated in a water bath at 37 ºC for 10 minutes to initiate enzyme inhibition. Thereafter, the residual plasma ChE activity in the reaction mixture was determined electrometrically as outlined above. All in vitro ChE inhibition assays were repeated four times for the baseline-control (0 μM) and dichlorvos concentrations at 0.25 and 0.5 μM, using the pooled plasma samples of the controls and the three RA groups of patients. The percentage of plasma ChE inhibition was estimated as follows:

% plasma ChE inhibition = [ChE activity in Δ pH/20 min (baseline control) - ChE activity (with dichlorvos)/ChE activity (baseline control)] × 100

Kinetic determination of in vitro plasma ChE inhibition by dichlorvos

Aliquots of plasma samples obtained from the controls and the three RA groups (10 each) were pooled separately to be used in this experiment using dichlorvos as an inhibitor [25,26]. The designated pooled plasma samples were incubated in vitro with dichlorvos at 0.25 μM for 5, 10, 15, 30, and 60 minutes, with the plasma ChE activity at 0-time without dichlorvos to be considered 100% baseline activity value [25]. Plasma ChE activity was determined at each time point in duplicate, and a suitable blank was included at each incubation time. Again, the residual plasma ChE activity after each incubation time was determined as mentioned above.

The in vitro decline of plasma ChE activity by dichlorvos vs. time (0 to 60 minutes) was used to apply steady-state kinetics for the following measurements as described in detail earlier [25]:

Log (plasma ChE activity) = Log (plasma ChE activity)0 - 0.434kt

Slope = 0.434k

- k = slope/0.434

Total inhibition time = 1/k

Inhibition rate= (plasma ChE activity)0 × k

where (plasma ChE activity) and (plasma ChE activity)0 were the enzyme activity at times of 60 and 0 minutes, respectively, with k as the inhibition rate constant. We also verified the results statistically by the linear regression analysis using the statistical software program Past4.13 (https://www.nhm.uio.no/english/research/resources/past/) and by the online program Omni Calculator (Chemistry Calculators, https://www.omnicalculator.com/chemistry).

Statistical analysis

We used the statistical software package PAST4.13 to statistically analyze data presented as mean ± SD by the F- and t-tests for two groups, and multiple groups by the one-way analysis of variance followed by Tukey’s multiple comparison test. The non-parametric Kruskal-Wallis test followed by the Dunn’s test was used to statistically analyze non-parametric data. Descriptive statistics were also used to characterize the data. The level of statistical significance was set at p<0.05.

Calculation of odds and risk ratios

Variations (usually 20% or more are significant) in ChE activity after drug or pesticide applications have been used to assess the risk of association of changes in ChE activity by odds and risk ratios [27,28]. Observed and expected cases of 20% increment in plasma ChE activity among RA patients were used to calculate odds and risk ratios by using PAST4.13. To achieve data categorization, an increase of 20% in plasma ChE activity (from a healthy-control value of 0.83 Δ pH/20 minutes) was taken into consideration. Accordingly, a table of odds and risk ratios of RA groups was constructed [27,28].

## Results

Demographic characteristics of RA patients

Table 1 presents the demographic characteristics of RA patients recruited for the study; the sample consisted of 76 (86.4%) females and 12 males (13.6%) while healthy controls included 22 (36.1%) females and 39 (63.9%) males. The RA patients were allocated into three groups according to the therapy they received regardless of the duration of the illness: no therapy 14 (15.9%), conventional therapy 49 (55.7%), and conventional therapy combined with biologic therapy 25 (28.4%). None of the patients received biologic treatment alone. According to the selection criteria, none of the patients had moderate to severe comorbidities. As shown in Table 2, the mean durations of RA illness in the three RA groups mentioned above were 3.14 ± 3.14, 10.18 ± 6.29, and 8.52 ± 5.99 years, respectively, and the duration of therapy in the last two groups varied considerably (51.90 ± 51.28 and 18.42 ± 22.08 months, respectively). The percentage of toxoplasmosis IgG positivity among controls and RA patients was 26.2% and 39.8%, respectively, and the difference was not statistically significant (p=0.087) (Table 3).

**Table 1:** Demographic characteristics of patients with RA and healthy controls BMI: body mass index; RA: rheumatoid arthritis; SD: standard deviation

Variable	RA patients (n=88)	Healthy controls (n=61)
Females, n (%)	76 (86.4%)	22 (36.1%)
Males, n (%)	12 (13.6%)	39 (63.9%)
Age, years, mean ± SD	44.61 ± 13.53	37.21 ± 9.91
BMI, kg/m^2^,mean ± SD	30.463 ± 7.554	30.90 ± 25.47
RA: no therapy, n (%)	14 (15.9%)	-
RA: conventional therapy, n (%)	49 (55.7%)	-
RA: conventional + biologic therapy, n (%)	25 (28.4%)	-

**Table 2:** Durations of RA illness and therapy in patients RA: rheumatoid arthritis; SD: standard deviation

RA groups	N	Duration of illness, years, mean ± SD	Duration of therapy, months, mean ± SD
RA: no therapy	14	3.14 ± 3.14	-
RA: conventional therapy	49	10.18 ± 6.29	51.90 ± 51.28
RA: conventional + biologic therapy	25	8.52 ± 5.99	18.42 ± 22.08

**Table 3:** Toxoplasmosis (IgG positivity) among patients with RA *Chi-squared test: 2.9353, p=0.087 (statistically significant) IgG: immunoglobulin G antibody; RA: rheumatoid arthritis

Groups	IgG-positive		IgG-negative	
	N	%	N	%
Healthy controls	16	26.2	45	73.8
Rheumatoid arthritis patients	35	39.8^*^	53	60.2

Plasma ChE activity in RA patients

**Figure 1:**
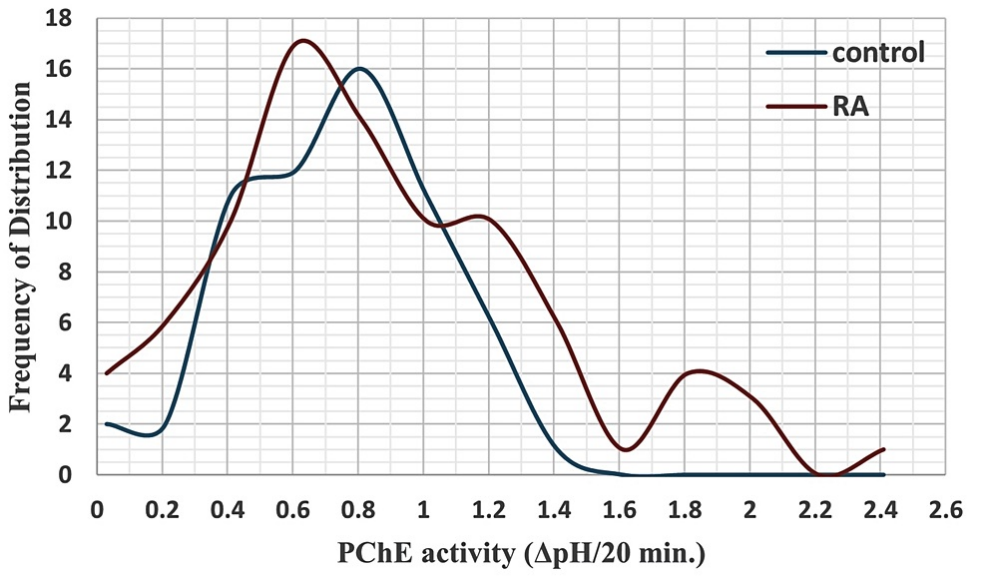
Distribution of plasma cholinesterase activity among healthy controls and patients with RA PChE: plasma cholinesterase activity; RA: rheumatoid arthritis

**Figure 2:**
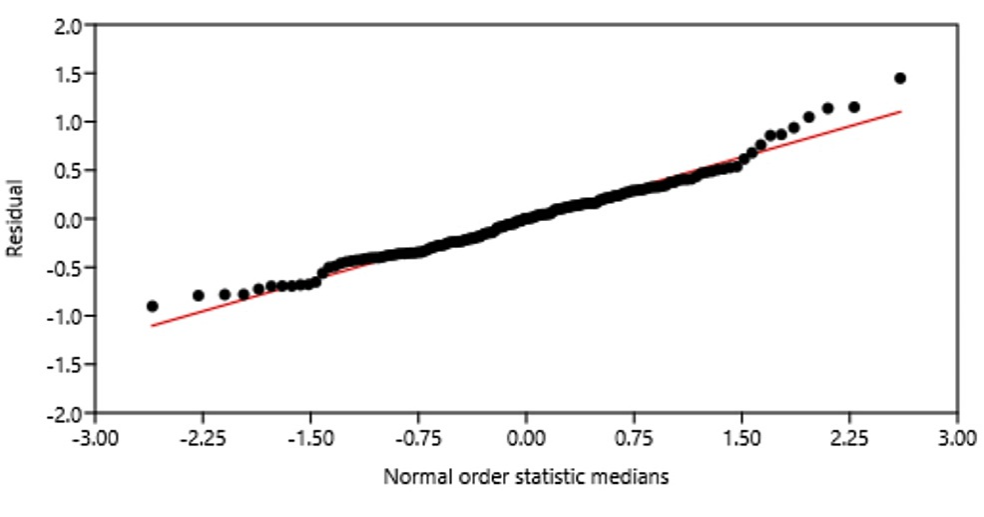
Normal probability distribution of plasma cholinesterase activity residuals of healthy controls and RA patients A total of 10 data points were deviated at both ends of the residual line RA: rheumatoid arthritis

As shown in Table 4, the mean plasma ChE activity of patients with RA was significantly higher than that of the control group, by 16%, and their variances were unequal (F-test: 1.6981, p same variance: 0.023724; t-test: -2.99, p same mean: 0.0032712). The plasma ChE values of both groups were not normally distributed (Figure 1), and due to this non-normal distribution, there were deviations of 10 data points that appeared on both ends of the residual distribution line (Figure 2). Further statistical analyses of plasma ChE values of RA subgroups revealed that the enzyme activities of the RA with conventional therapy and RA with conventional + biologic therapy were higher than that of the control group, by 18% and 27%, respectively (Table 5). The plasma ChE activity of the RA-no therapy group was lower than that of other RA therapeutic groups, with a statistically significant difference in the third RA group (Table 5).

**Table 4:** Plasma cholinesterase activity in patients with RA in comparison with healthy controls *Significantly different from the control group value at p<0.05 (one outlier was detected in the rheumatoid arthritis group) RA: rheumatoid arthritis; SD: standard deviation

Parameter	Healthy controls	RA patients
N	61	88
Cholinesterase activity, Δ pH/20 min, mean ± SD	0.83 ± 0.30	0.96 ± 0.50^*^
F test	2.697	
P-value (same variance)	<0.0001	
t-test	2.0494	
P-value (same mean)	0.04225	

**Table 5:** Plasma cholinesterase activity in patients with RA *Significantly different from the RA-no therapy groups at p≤0.05, Krukall-Wallis test: 6.632, p=0.08455, followed by Dunn’s test at p≤0.05 ChE= cholinesterase; RA= rheumatoid arthritis; SD: standard deviation

Groups	M	ChE activity, Δ pH/20 min, mean ± SD	ChE activity, Δ pH/20 min, median (25–75 percentile)	P-value, Dunn’s test, the control group	P-value, Dunn’s test, the RA-no therapy group
Healthy controls	61	0.83 ± 0.30	0.87 (0.61–1.08)	-	0.2867
RA-no therapy	14	0.71 ± 0.30	0.76 (0.555–0.88)	0.2867	-
RA-conventional therapy	49	0.98 ± 0.56	0.92 (0.58–1.345)	0.2903	0.08704
RA-conventional + biologic therapy	25	1.05 ± 0.44	1.05^*^ (0.71–1.275)	0.05658	0.02133^*^

We constructed the table illustrating the odds ratio for the three RA groups, taking into account the possibility of elevation in plasma ChE activity (risk cases of high ChE) in the RA-conventional therapy (group 2) and RA-conventional therapy + biologic therapy (group 3) vs. RA-no therapy (Group 1) by 20% (>0.83 Δ pH/20 min of the control value to be ≥1.0) (Table 6). Afterward, estimating the odds ratio and the risk ratio of occurrence of such an elevated plasma ChE activity resulted in odds ratio and risk ratio values of 9.75 and 6, respectively in group 2, and 16.55 and 7.84, respectively in group 3 (Table 7). These values indicated an association in terms of the occurrence of cases of high plasma ChE activity within the RA patients subjected to conventional therapy alone or in combination with biologic therapy.

**Table 6:** Odds ratio table* *The table illustrates the frequency of the occurrence of plasma cholinesterase activity ≥1.0 Δ pH/20 min** in patients with RA treated with conventional therapy or conventional + biologic therapy compared to the RA-no therapy group **An increase of 20% in ChE activity (healthy control: 0.83 Δ pH/20 min) was considered a risk factor ChE: cholinesterase; RA: rheumatoid arthritis

Groups	ChE ≥ 1.0 Δ pH/20 min	ChE < 1.0 Δ pH/20 min	Total
RA-conventional therapy	21	28	49
RA- conventional + biologic therapy	14	11	25
RA-no therapy	1	13	14

**Table 7:** Results of odds and risk ratios* *The table illustrates the results of odds and risk ratios of a 20% increase in plasma cholinesterase activity among patients with RA treated with conventional therapy or conventional + biologic therapy compared to the RA-no therapy group RA: rheumatoid arthritis

Group	Odds ratio (95% confidence interval)	p (ratio=1)	Risk ratio (95% confidence interval)	p (ratio=1)
RA-conventional therapy	9.75 (1.181, 0.52)	0.035	6 (0.883, 40.77)	0.067
RA-conventional + biologic therapy	16.55 (1.867, 46.6)	0.012	7.84 (1.15, 53.5)	0.036

Plasma ChE activity in toxoplasma IgG-positive RA patients

Statistical comparison of plasma ChE activity of toxoplasma IgG-positive and IgG-negative cases among patients with RA as well as control participants did not reveal any significant difference (Table 8).

**Table 8:** Plasma cholinesterase activity in toxoplasma IgG-positive and negative patients with RA ChE: cholinesterase; RA: rheumatoid arthritis; SD: standard deviation

Group	N	ChE activity, Δ pH/20 min				P-value
		Toxoplasma IgG-positive, mean ± SD	N	Toxoplasma IgG-negative, mean ± SD	N	
Healthy controls	61	0.799 ± 0.303	16	0.847 ± 0.303	45	0.587
RA-no therapy	14	0.760 ± 0.217	8	0.637 ± 0.390	6	0.508
RA-conventional therapy	49	1.088 ± 0.555	17	0.925 ± 0.559	32	0.336
RA-conventional + biologic therapy	25	1.055 ± 0.380	10	1.047 ± 0.482	15	0.962
Overall	149	0.939 ± 0.425	51	0.890 ± 0.437	98	0.509

In vitro ChE inhibition

In vitro exposure of plasma samples of controls and RA patients to dichlorvos at 0.25 and 0.5 μM significantly and in a concentration-dependent manner inhibited the plasma ChE activity compared to their corresponding baseline-control (0 μM dichlorvos) values (Table 9). The rates of plasma ChE inhibition by dichlorvos at 0.25 and 0.5 μM in the control group were 77% and 91%, respectively, and in the three RA groups, they were as follows: RA-no therapy (73% and 89%), RA-conventional therapy (56% and 71%), and RA-conventional therapy + biologic therapy (41% and 53%), respectively (Table 9).

**Table 9:** In vitro inhibition of plasma cholinesterase activity by dichlorvos among healthy controls and patients with RA Pooled plasma (of 10 individuals/group) ChE values are presented as mean ± SE of four determinations/each dichlorvos concentration *Significantly different from the baseline (0 μM) concentration (p<0.05) ChE: cholinesterase; RA: rheumatoid arthritis

Dichlorvos, μM	ChE activity, Δ pH/20 min	% inhibition	P-value
Healthy controls			
0 (baseline)	0.56 ± 0.058	-	-
0.25	0.13 ± 0.023^*^	77	0.000039
0.5	0.05 ± 0.008^*^	91	0.0000097
RA-no therapy			
0 (baseline)	0.41 ± 0.016	-	-
0.25	0.11 ± 0.026^*^	73	0.000012
0.5	0.05 ± 0.022^*^	89	0.0000023
RA-conventional therapy			
0 (baseline)	0.34 ± 0.075	-	-
0.25	0.15 ± 0.016^*^	56	0.0348
0.5	0.10 ± 0.014^*^	71	0.0104
RA- conventional + biologic therapy			
0 (baseline)	0.57 ± 0.077	-	-
0.25	0.34 ± 0.029^*^	41	0.0296
0.5	0.27 ± 0.038^*^	53	0.0067

Kinetics of in vitro plasma ChE inhibition

In all groups (controls and RA), plasma ChE activity declined progressively in response to incubation times of zero to 60 minutes with dichlorvos at 0.25 μM (Table 10). The percentage decreases of plasma ChE activity among the four groups from the respective zero time value (100%) to 60-minute values were 93.4%, 89.4%, 88.1%, and 89.4%, respectively (Table 10). However, despite the similarity in these final inhibition percentages, estimation of the kinetic values derived from the steady state equation, Log (plasma ChE activity) = Log (plasma ChE activity)0 - 0.434 kt, revealed that the RA groups showed increases in the half-life of inhibition (t1/2) by 23.6% to 32.7% and the total inhibition time by 23.5% to 32.5% (Table 11). These changes coincided with decreases in the inhibition rate constant (k) among the RA groups by 19% to 24.5%, an indication of the reduced inhibition rate of plasma ChE activity compared to that of the control group (Table 11).

**Table 10:** Progression of in vitro inhibition of plasma cholinesterase activity by dichlorvos (0.25 μM) at different incubation times among healthy controls and RA patients Values are presented as the mean of duplicate ChE measurements at each time point in 10 pooled plasma samples/group ChE: cholinesterase; RA: rheumatoid arthritis

Time (minutes)	ChE activity, Δ pH/20 min			
	Healthy controls	RA-no therapy	RA-conventional therapy	RA-conventional + biologic therapy
0	0.915	0.895	1.135	0.855
5	0.445	0.495	0.615	0.46
10	0.35	0.39	0.55	0.42
15	0.345	0.375	0.405	0.335
30	0.15	0.235	0.275	0.165
60	0.06	0.095	0.135	0.095

**Table 11:** Time-dependent kinetic parameters of plasma cholinesterase inhibition among healthy controls and RA patients following in vitro incubation of 10 pooled plasma samples with dichlorvos at a concentration of 0.25 μM *Percentage values represent changes from the corresponding control value The data from Table 10 were used to calculate time-dependent kinetic parameters of ChE inhibition ChE: cholinesterase; RA: rheumatoid arthritis

Groups	Inhibition rate constant (k), min^-1^	Half-life of inhibition (t_1/2_), min	Inhibition rate, ChE activity Δ pH/ min	Total inhibition time, min
Healthy controls	0.042	16.5	0.038	23.81
RA-no therapy	0.033	20.8	0.03	30.03
% change^*^	–21.4	26.1	–21.1	26.1
RA-conventional therapy	0.0317	21.9	0.036	31.546
% change	–24.5	32.7	–5.3	32.5
RA-conventional + biologic therapy	0.034	20.4	0.029	29.412
% change	–19.0	23.6	–23.7	23.5

## Discussion

During the 16-month research period, females constituted a significant majority of the RA patients recruited to the study (86.4%), which aligns with other studies globally [3] or locally in Iraq [28,29]. The no-therapy group of RA patients comprised only 14 (15.9%) of the total 88 RA patients. The low presence of this group was expected, as it is hard to recruit RA patients who had not been treated previously since their well-being is dependent on the implementation of therapy among other factors that affect the progress of the illness [29,30].

The toxoplasma IgG positivity in the RA patients in the present study was 39.8% vs. 26.2% among controls. This finding is consistent with the findings of a meta-analysis in which the percentage of toxoplasmosis positivity in RA patients was 46% vs. 21% among controls [12]. Another reported a value of 48% in RA patients vs. 10% in controls [11]. The pathogenesis of toxoplasmosis in exacerbating the conditions of RA patients is still obscure; however, this zoonotic disease is opportunistic in nature and causes infection in immunocompromised patients, as is the case with RA [12,13].

The plasma ChE activity of RA patients was significantly higher than that of the control group, by 16%. This is a unique finding compared to previous reports, which indicated that ChE activity in the blood, mainly that of the erythrocytes, can be modulated by the RA disease process [16,17]. It has been found that high plasma ChE activity and cholinergic anti-inflammatory pathway could be implicated in slowly developing inflammatory disease, as is the case with RA [15,18]. The clinical implication of the present finding is, however, not clear yet. Further examination of plasma ChE activity according to RA therapy groups suggested that high enzyme activity could be related to both conventional and biologic therapy (Table 5). However, the high level of variability in the durations of the illness and RA therapy might have precluded the finding of a clear-cut cause-effect relationship between therapy and plasma ChE activity. Nevertheless, considering the increased plasma ChE activity (≥20%) in the RA patients as a risk factor, we detected an association in terms of the occurrence of cases of high enzyme activity among RA patients undergoing conventional therapy alone or in combination with biologic therapy. The role of the therapeutic agents, whether conventional or biologic, given for a long period, in modulating plasma ChE activity is not clear at present. They might, however, increase the output of the enzyme by the liver, which is the target organ for drug biotransformation as found in many slowly developing inflammatory diseases [15,16,18].

We took another step in examining the plasma ChE activity of RA patients by the in vitro challenging technique with the potent ChE inhibitor dichlorvos. As Expected [25,26], dichlorvos inhibited the enzyme activity in the control and RA groups. However, it was clear that the plasma ChE in the two RA therapy groups (conventional alone or with biologic agents) was less susceptible to inhibition by 20% to 38% (Table 9). Additional experimental support for the possibility of RA-modulated ChE activity in the present study was obtained from the inhibition kinetics experiment (Tables 10-11). This experiment indicated increased half-life of inhibition and total inhibition time concurrently with decreased inhibition rate constant (k) among the RA groups. These kinetic changes collectively support and reveal the reduced susceptibility of plasma ChE activity among the RA patients to the in vitro dichlorvos inhibition (Tables 9-11). Further studies are needed to more thoroughly explore the finding of reduced plasma ChE susceptibility to inhibition, and determine whether this could be an added advantage or even a disadvantage in RA patients receiving other therapeutic ChE inhibitors, as is the case among those additionally inflicted with Alzheimer’s disease.

The concurrent burden of toxoplasmosis (IgG-positive cases) among RA patients in the present study was not a risk factor in affecting plasma ChE activity. However, acute *T. gondii* infection of experimental animals revealed increases [19-21] or even decreases [22] in plasma or tissue ChE activity, probably by causing liver injury or affecting the cholinergic anti-inflammatory pathway [18]. It is not conceivable to conclude a lack of anti-ChE effects of toxoplasmosis among RA patients in the present study. This is due to the unknown effects of contributing factors that might impact the enzyme activity, such as the chronic nature of both diseases, obscure timing of toxoplasmosis infection, as well as the dilemma of the RA medications in modulating the ChE activity in tissues or the blood.

Limitations of the study

In the present study, we could not assess the correlation of plasma ChE activity with the duration of RA signs and symptoms because the patients were not aware of the nature of the disease and they did not consult the specialists until it became painful and affected their quality of life. The effects of RA medications, usually more than one agent, on plasma ChE activity could not be verified solely. The role of concurrent toxoplasmosis with RA in modulating plasma ChE is not clear from the present findings, which could be due to the nature of disease complications and/or multiple therapies.

## Conclusions

The autoimmune nature of RA and its chronicity might have contributed to the increase in plasma ChE activity among the patients. This increase in enzyme activity could be a risk factor in RA patients undergoing conventional therapy alone or in combination with biologic therapy; however, the clinical significance of increased ChE activity remains obscure at present. Nevertheless, the in vitro inhibition experiments on plasma ChE activity of RA patients suggest reduced susceptibility of the enzyme to ChE inhibition by dichlorvos, which calls for in-depth explorations of adverse or even beneficial effects of other ChE inhibitors that could be applied in RA patients undergoing anti-arthritic therapy. Toxoplasmosis being an opportunistic infection was not a risk factor when plasma ChE activity was taken into account among RA patients. However, the prevalence of toxoplasmosis was expectedly high among RA patients.
